# Enhanced morphological and physiological responses of micro-propagated cassava through arbuscular mycorrhizal fungus inoculation

**DOI:** 10.3389/fpls.2026.1692288

**Published:** 2026-03-09

**Authors:** Ika Wahyuni, Bolaji Thanni, Achmad Subagio, Herve Vanderschuren, Roel Merckx, Stefan Hauser, Olivier Honnay

**Affiliations:** 1 Department of Biosystems, Crop Biotechnics, Katholieke Universiteit (KU) Leuven, Heverlee, Belgium; 2 Department of Earth and Environmental Sciences, Division Soil and Water Management, Katholieke Universiteit (KU) Leuven, Leuven, Belgium; 3 Department of Biology, Agronomic and Conservation Ecology, KU Leuven, Leuven, Belgium; 4 Root and Tuber Agronomy, International Institute of Tropical Agriculture, Ibadan, Nigeria; 5 Department of Agroindustrial Technology, University of Jember, Jember, Indonesia; 6 KU Leuven Plant Institute (LPI), KU Leuven, Leuven, Belgium; 7 Plant Genetics and Rhizospheric Processes Laboratory, Gembloux Agro BioTech, University of Liège, Gembloux, Belgium

**Keywords:** acclimatization, cassava, drought, micropropagated plant, *Rhizophagus irregularis*, AMF

## Abstract

Micropropagation is instrumental for the rapid multiplication of elite cassava varieties with improved traits. However, it is often impaired by transplanting stress associated with acclimatization, which occurs during the acclimatization, transfer from *in vitro* to soil condition. Arbuscular Mycorrhizal Fungi (AMF) could be used to reduce transplant shock symptoms through improved nutrient acquisition and physiological function. In this study, we investigated the impact of inoculating anAMF, *R. irregularis*, on the growth of two varieties of micropropagated cassava on plant physiological traits, the δ ^13^C and δ ^15^N isotopic signatures, and nitrogen use efficiency (NUE) during acclimatisation. In the early acclimatization stage, (4–8 weeks after transplanting,WAT), AMF inoculation increased height, stem diameter, and leaf number by 61%, 50%, and 57% respectively, compared to non-inoculated plants in non-sterile soil. Under subsequent water deficit at 8–10 WAT, inoculated plants were better preserved regarding height and stem thickness. After 10 weeks, AMF root colonization increased by 57% and 61% under water-deficit and well-watered conditions respectively, compared to non-inoculated plants. Total biomass, C:N and NUE increased significantly in inoculated plantlets under well-watered conditions, with lowered δ ^15^N and N concentrations due to N dilution from a 63% biomass increase. In water-deficient conditions, regardless of inoculation, δ ^13^C decreased, suggesting the dominant role of water availability in carbon assimilation. Overall, we found important benefits of AMF inoculation. Yet, to fully harness the benefits of AMF for micropropagated cassava, proper nitrogen management is essential, as the enhanced growth conferred by increased root colonization rate could lead to N deficiency.

## 1 Introduction

Cassava (*Manihot esculenta* Crantz) is a tuberous root crop cultivated in tropical and subtropical areas and is usually planted from stem cuttings. However, to avoid the transfer of diseases, using plant tissue-culture techniques to replicate and grow cassava can be a good alternative as it efficiently generates a significant quantity of genetically identical plantlets free from pests and diseases (Ilan and Woldetsadik, 2011; Raaman and Patharajan, 2006). A significant limitation of the micropropagation technique is the high mortality rate of *in-vitro*-raised plants during and after their transfer from the laboratory to the greenhouse, posing a significant barrier to its widespread use (Hasnain et al., 2022). Even under optimal conditions, only about 78% of cassava plantlets typically survive during acclimatization (Kidulile et al., 2018). Mortality remains attributed mainly to inadequate root system development, low photosynthetic capacity, both of which restrict growth and establishment (Grout and Aston, 1978; Grout and Millan, 1985). This figure still implies that more than one in five plantlets fail to establish. In large-scale propagation programs, such attrition translates to substantial time, cost, and labor losses. Furthermore, these figures are often reported under controlled, ideal conditions. Under real-world scenarios, especially in resource-limited or drought-prone regions, survival rates may be significantly lower, highlighting the urgency to improve robustness and resilience during post-acclimatization.

Acclimatization and post-acclimatization are two important stages for a successful plantlet transition. During acclimatization, plants are potted into peat moss or perlite substrate, often coupled with plastic covering for the pots to maintain high humidity and limit moisture loss (Solangi et al., 2022). Acclimatization is a crucial phase, as plantlets exhibit weak stomatal control coupled with high cuticular water loss, making them highly vulnerable to abiotic stress (Santamaria et al., 1993). Early death is generally credited to variable high humidity or excessive water availability of the substratum (particularly for sensitive genotypes), suboptimal in nutritional conditions, which together makes plants highly vulnerable during acclimatization (Pasternak and Steinmacher, 2025). However, work related to the phase of post-acclimatization remains scant.

After acclimatization, micropropagated cassava plantlets will have passed onto fully independent autotrophic growth, during which they are likely to be influenced negatively by environmental stresses, with water deficit being one of the significant issues affecting them (Abahmane, 2011; Abdul-Soad and Al-Khayri, 2018). As climate instability increases in cassava-growing regions and cultivation expands into more drought-prone areas, understanding plant responses to drought during the post-acclimatization phase is becoming increasingly important. Enhancing cassava survival during this stage will improve micropropagation efficiency and support cassava production under changing climatic conditions (Feyisa, 2021; AGROSAVIA, 2023).

Drought stress during post-acclimatization can hinder normal plant growth and development by disrupting photosynthesis and carbon metabolism. In response to water deficit, plants undergo morphological and physiological changes, including stomatal closure, reduced CO_2_ uptake, and decreased photosynthetic activity (Cornic and Massacci, 1996). These physiological constraints are responsible for reducing the establishment and productivity of plants (Chaves et al., 2009; Flexas et al., 2006). Carbon isotope values (δ¹³C), which integrates long-term changes in stomatal conductance and carbon fixation, provides a useful proxy in assessing plant response to water availability (Farquhar et al., 1989). While the concept of δ¹³C has been extensively explored for drought stress response in field-grown cassava, its role in micropropagated cassava under greenhouse conditions is yet to be explored.

AMF possesses a potential approach for increasing survival chances for water scarcity post-acclimatization. Through their symbiotic relation with roots, AMF enhances the phosphorus and other minerals uptake and may influence plant growth regulators, thereby strengthening establishment and overall stress tolerance (Smith and Read, 2008; Van Geel et al., 2016). Although other PGPM (Plant Growth-Promoting Microorganism), like rhizobacteria or endophytic fungi, have been evaluated in cassava, the primary relevance of AMF is in its direct association with enhancing water acquisition and, thereby, drought adaptational ability (Johansson et al., 2004; Köhl et al., 2016). AMF relieve transplant shock and extreme environmental variation by optimizing root development and regulating stomata functions and nutrient uptake (Cheng et al., 2002; Hu et al., 2017). AMF-assisted acclimatization has been extensively examined in other micropropagated crops such strawberries (Elmeskaoui et al., 1995) and pepper (Estrada-Luna and Davies, 2003) yet remains scarcely studied in cassava (Azcón-Aguilar and Barea, 1997). Although much emphasis has been placed on improving acclimatization success, little is known about how micropropagated cassava responds to environmental stress during the subsequent post-acclimatization phase, which is critical for field establishment.

The overall objective of this study was to test whether the application of AMF enhances the morphological and physiological characteristics of micropropagated cassava during and after acclimatization under greenhouse conditions. Two cassava varieties, representing contrasting genetic backgrounds or physiological responses, were selected to evaluate whether the beneficial effects of AMF are consistent across genotypes or vary with genetic diversity. To guarantee the broader applicability of our results, the experiment was performed on non-sterilized soil. Specifically, we aimed to:

1. Assess the effect of *Rhizophagus irregularis* on the vegetative growth of micropropagated cassava during acclimatization;
2. Explore the effect of *Rhizophagus irregularis* on photosynthetic capacity and the carbon (C) and nitrogen (N) nutritional composition of micro-propagated cassava plantlets under well-watered and water-deficient conditions during the post-acclimatization phase in non-sterilized medium;and
3. Examine the δ¹³C and δ¹^5^N signature as indicators of nitrogen uptake, C:N balance and nitrogen use efficiency (NUE).

## 2 Methods

### 2.1 Experimental design and treatments

The experiment consisted of a full factorial 2 × 2 × 2  randomized control trial with three factors: (1) two AMF inoculum levels - inoculation with *Rhizophagus irregularis* (+ inoculum) and an un-inoculated control (- inoculum); (2) two water regimes - well-watered (WW) at 100% pot capacity and water deficit (WD); and (3) two cassava varieties - TME 419 and cv. 60444. These treatments were arranged in a completely randomized design, with 4 replications, totaling 32 experimental pots.

### 2.2 Study site and planting medium

The study was carried out in the greenhouse at KU Leuven, Belgium, with a maximum of 14 hours of light, 50-60% humidity, and day/night temperatures of respectively 25 °C and 20 °C.  The planting medium was a mix of natural loamy soil collected from the agricultural Ter Munck field, Leuven, and quartz sand (<2mm) at 3:1 v/v. The properties of the loamy soil were: pH of 6.7, 0.9% organic C, 59 mg/L available P (Olsen), and 0.1% total N.

### 2.3 Biological material and plant cultivation

#### 2.3.1 Inoculum preparation

*Rhizophagus irregularis* was chosen for the experiment based on its dominance across cassava fields in Nigeria (Thanni et al., 2022). The isolate was purchased from INVAM (International Culture Collection of (Vesicular) Arbuscular Mycorrhiza Fungi), West Virginia University, USA. Multiplication of the AMF inoculant was done using cassava as a host plant for three months in a greenhouse using a sterilized silica-vermiculite substrate consisting of white silica sand (70%) and vermiculite (30%). After 3 months, watering was stopped for a week and the roots were taken out and cut into pieces (< 5 mm) before mixing them with the entire silica-vermiculite substrate.  The inoculants (a mix of roots and substrate) contained no other soil microbiota except for inoculated *Rhizophagus irregularis* and were stored at 4°C for several weeks before use.

#### 2.3.2 Cassava plantlets multiplication via subculturing and AMF inoculation

*In vitro* regenerated plantlets of cassava varieties cv. 604444 and TME-419 were provided by Gembloux Agro-Bio Tech, University of Liège. To obtain enough plantlets for the experiment, the nodal segments (5–10 cm) from the *in-vitro* regenerated plantlets were prepared and sub-cultured onto cassava basic *in vitro* medium (CBM) following Bull et al. (2009), supplemented with 20 g/l sucrose and 1 ml of 2mM CuSO4 using sterile disposable plastic jars. The pH of the medium was adjusted to 5.8 and 2.5 g L^−1^ Phytagel™ (Sigma-Aldrich^®^) was added for gelling per liter of medium. Media were autoclaved for 1 hour at 120 °C and 115 kPa according to the protocol (Murashige and Skoog, 1962; Bull et al., 2009). The explants were incubated at 24 ± 2 °C, 40 ± 5 μmol m^−2^ s^−1^ irradiance, at 16-h photoperiod. At 4 weeks after incubation, well-rooted plantlets of homogenous sizes were taken from the jars, root washed and transplanted immediately into polypropylene trays withthe same non-sterilize loamy soil – sand mixture (3:1, v/v). The inoculation treatment consisted of adding 5 g of the AMF inoculant (mix of roots and soil substrate) to each planting hole. To forestall the risk of low colonization and to attain 500 spores per planting hole. Control plants did not receive a sterilized inoculum because our experiment wanted to mimic realistic conditions. Given that the used natural soil contained organic matter and that all treatments further provided with sufficient nutrients. The total nitrogen (0.083%), Olsen phosphorus (2.54 mg/kg), and total organic carbon (<0.3%) contents were found to be low. Exchangeable cation contents were also low: calcium (1.88 meq/100 g), potassium (0.147 meq/100 g), magnesium (3.34 meq/100 g), and sodium (0.113 meq/100 g). At the application rate of 5 g inoculum in 5 kg planting medium (0.1% w/w), the nutritional contribution of the inoculant is considered to be negligible, justifying its use as a carrier of AMF propagules without influencing soil fertility in any physical way and the sterilized inoculum was not needed on the experiment.

The pots containing plantlets were placed on plastic tray, and water was added to the tray until the pots were partially submerged, saturating the substrate by capillarity. Once the substrate was fully saturated, the pots were removed from the water. Afterwards, the plantlets were fully covered with a transparent lid in the greenhouse at KU Leuven with 12 hours of light, and 60% relative humidity. After a week, 60 ml of the Hoagland solution (1 M Ca (NO_3_)_2_ × 4H_2_0; 1 M KNO_3_; 1M MgSO_4_; 1 M KH_2_PO_4_; 1 M KCL; 8.96 mM Fe) and other micronutrients) and applied as fine spray using a wash bottle. Plantlets were kept in the greenhouse with a half-opened lid for another week. The total volume of Hoagland solution sprayed per plant was recorded for subsequent NUE calculation.

### 2.4 Post-acclimatization and introduction of water stress

Six-week-old plantlets were transplanted to the 5L pots filled with the same non-sterilized loamy soil and quartz sand mix. Pots were placed on a bench in the greenhouse and the pot’s positions were switched every morning to control for potential position effects. To avoid possible cross contamination between inoculated and the uninoculated treatment, all the pots that received AMF inoculum were placed together. All pots were maintained at 100% PC (pF 2, ± 0.2 cm^3^/cm^3^ VWC) until 60 days after planting (DAP). This was achieved by weighing the pots and adding the amount of water lost on the previous 2 days. By the 61^st^ DAP, watering was withdrawn from the pots marked for drought and this continued till the water content reached pF 3.3, ± 0.07 cm^3^/cm^3^ VWC on the 73^rd^ DAP.

### 2.5 Plant growth and biomass accumulation

Plant height and stem diameter were determined using a ruler and Vernier caliper. Measurements were taken three times at 1-week intervals starting from a week before the water deficit (WBD) was imposed till the 2^nd^ week after the deficit (1 WAD and 2 WAD) was introduced. The number of emerging leaves was counted. Following the directions in the user manual, stomatal conductance was assessed using the fourth fully expanded leaves between 9 and 11 am at 1 and 2 WAD using an SC-1 Degacon porometer device. Harvesting was carried out at 73 DAP. The biomass of shoots and roots were measured after oven-drying the samples at 70 °C for 48 h.

### 2.6 Isotopic analyses, leaf chlorophyll concentration, percent C and N, and NUE

The youngest fully expanded leaves (YFEL) were collected for stable isotope and leaf chlorophyll determination at harvest. The YFEL collected from the plants were oven-dried at 70°for 48 h and finely ground in a ball mill. The samples were analyzed for δ^13^C and δ^15^N isotopes using an elemental analyzer (Thermo Flash HT/EA or EA 1110) coupled to an IRMS system (Thermo Delta V Advantage) via a Conflo IV interface from Thermo. The final delta values are expressed relative to international standards VPDB (Vienna PeeDee Belemnite) for δ^13^C and atmospheric N_2_ for δ^15^N. The total C and N concentrations of the YFEL were also measured using the elemental analyzer.

Leaf chlorophyll concentration was determined from 0.5 g of the YFEL leaf submerged in 80% acetone for 48 hours. The optical density of the extract was measured at 663 nm and 645 nm wavelengths, using the Evolution 201 UV-visible Spectrophotometer (Thermo Scientific, Waltham, MA, USA). The total amount of chlorophyll (mg/g) was calculated following the equation: (0.00802 × A_663_ + 0.0202 × A_645_)v/w as described by Yoshida et al. (1976), where A_663_ and A_645_ are the absorbances at 663 nm and 645 nm wavelengths, *v* the final volume of chlorophyll extract and  the fresh weight of leaf tissue extracted.

For the nitrogen use efficiency (NUE), we first calculated the N*input* (**Equation 1**), we estimated the N*input* per plant based on the concentration in the sprayed Hoagland solution and the total amount sprayed, and then the NUE was calculated using **Equation 2**.

(1) $${\text{N}}_{input}=\text{C}\times \text{V}$$

Where C= concentration of nitrogen in Hoagland solution; V= volume of the Hoagland solution sprayed.

(2) $$NUE=\frac{W}{Ninput\;}\;\times 100$$

Where N*_input_* = total nitrogen input; W= total biomass.

### 2.7 Root colonization by modified ink staining 

At harvest, a portion of the roots (1–2 g) was carefully washed and cut into 2 cm long segments. Root segments were cleared with 5% KOH at 60 °C for 40 minutes and rinsed with tap water for acidification with 1% HCl at room temperature for 5 minutes. The segments of the roots were then stained with 61% ink solution in 100 ml vinegar for 1 hour at 90 °C (Sylvia, 1994). The rate of mycorrhizal colonization was determined using the gridline intercept method described by McGonigle et al. (1990). The presence of hyphae, coils, and vesicles was considered as evidence of mycorrhization and was used to estimate colonization intensity.

### 2.8 Statistical analyses

An analysis of repeated measurements was conducted using a Linear Mixed Effect Model (LMER) in R version 4.2.2, accounting for the non-independence of the multiple measurements taken on the same sample for plant height, stem diameter, number of leaves and stomatal conductance. For the parameters collected at harvest; shoot and root biomass, total chlorophyll content, δ^13^C and δ^15^N a LMER model was used. AMF inoculation (inoculum +/-), water regime (WW/WD), variety (TME 419/cv. 604444), and their interactions were also incorporated into the model as fixed factors, and the replicate was employed as a random factor. Data were first examined for normality and homogeneity of variance using the Shapiro-Wilk and Levene’s tests, respectively. These diagnostics were used to verify that residuals met the normality and homoscedasticity assumptions of the model. The lmer function in the “lme4” package was used to run the model (Bates et al., 2015), and the anova function in the “lmerTest” package was used to evaluate the significance of the test (Kuznetsova et al., 2017). Data on root colonization were analyzed using a generalized linear mixed model, following a Poisson distribution. To reveal the key parameters involved in the response patterns of micro-propagated cassava to AMF inoculation and water regimes, a Factor Analysis of Mixed Data (FAMD) was performed using data of morphological and physiological parameters related to growth, biomass accumulation, photosynthesis, stable isotope ^13^C and ^15^N, total C and N and NUE. The *FactoMineR* (Lê et al., 2008) and *factoextra* (Kassambara and Mundt, 2020) packages in the R software were used for the multivariate analysis and visualization of results.

## 3 Results

### 3.1 AMF colonization

**Figure 1:**
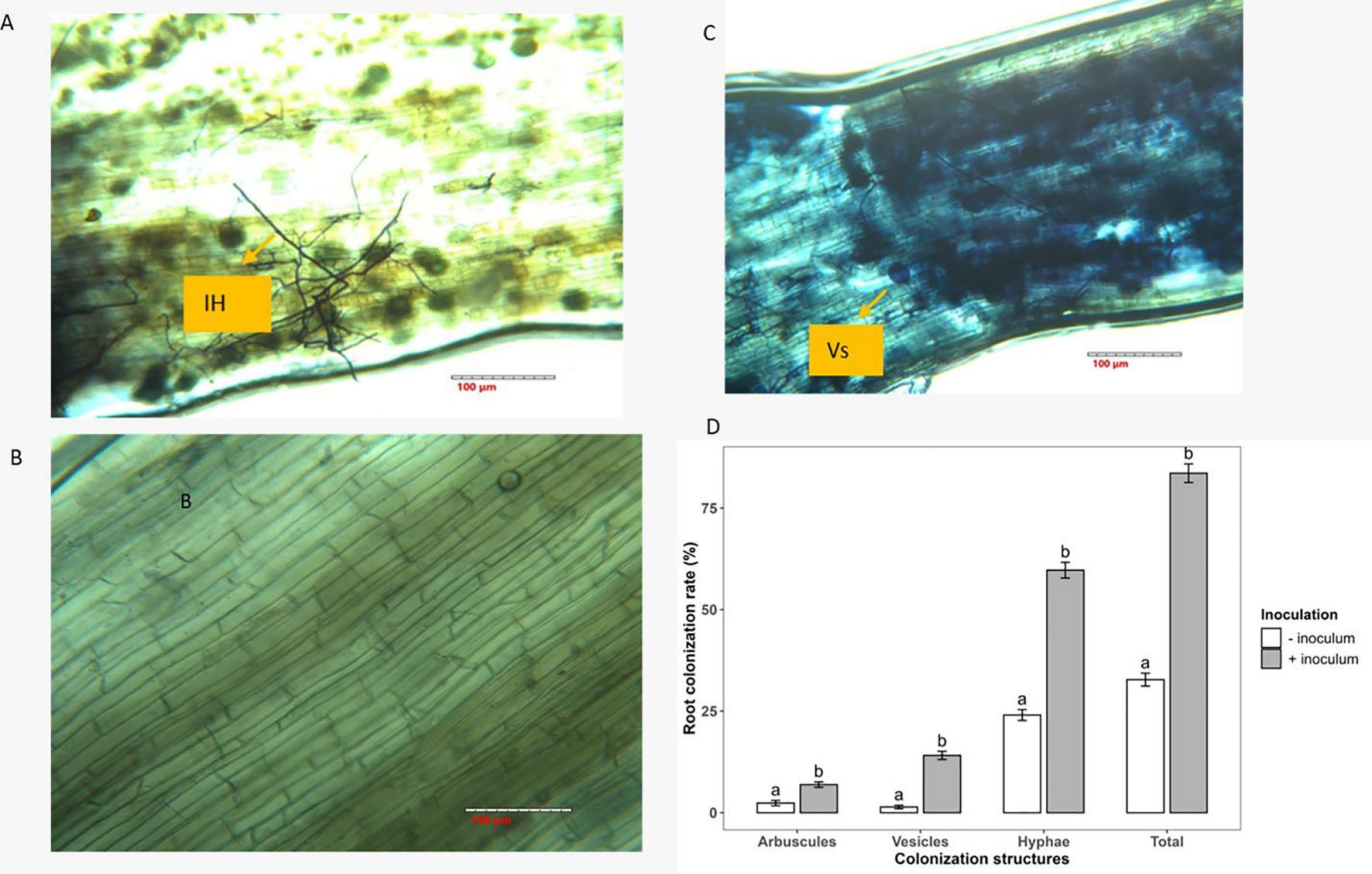
Colonization of arbuscular mycorrhizal fungus in **(A)** Cassava plantlets inoculated with commercial *R. irregularis* and **(B)** uninoculated cassava plantlets**. (C)** Vesicles in cassava inoculated with commercial *R. irregularis***(D)** Colonization rate of cassava root under inoculated and uninoculated conditions. **(A, C)** bars = 100 μm and **(B)** bars = 40 μm. Ih, intraradical hyphae; Vs, vesicles; Ar, arbuscules; Pc, plant cell; NAM, non-AMF-inoculated. Different lowercase letters above the bars indicate significant differences (*p* < 0.05) among inoculation treatments. Values are emmeans ± SE. IH, intraradical hypha; Vs, vesicles.

The roots of the + inoculum cassava plantlets at harvest clearly showed a high proliferation of AMF hyphae (**Figure 1**). - Inoculum plants showed negligible colonization (<5%) which only consist of hyphae, confirming minimal background AMF presence in the soil. The hyphae, vesicles and arbuscules were increased (p<0.001) by 59.7%, 90.1% and 65.4%, respectively, compared with the uninoculated plants (**Supplementary Table S1**; **Table 1**). Water deficit significantly reduced the presence of vesicles (p<0.001), and arbuscules (p<0.01) by 50.2% and 74.9%, respectively, but did not affect the hyphae. Vesicle and arbuscule formation in the plantlet roots was different between the varieties; cv. 604444 had more vesicles (8.14 ± 1.49) compared with TME 419 (2.43 ± 0.79) while TME 419 (6.43 ± 0.71) had more arbuscules than cv. 60444 (2.54 ± 0.71). Furthermore, there was a significant three-way interaction of inoculation × water regime × variety (p ≤ 0.05; **Table 2**) such that inoculation of cv. 60444 increased the number of hyphae in the well-watered condition by 61.5% over the hyphae in the roots of the well-watered uninoculated plants. In TME 419, inoculation increased the number of hyphae by 50.0% in well-watered plants compared with well-watered uninoculated plants. Under water deficit conditions, inoculation increased the number of hyphae by 39.3% in cv.60444 and by 67.8% in TME 419 when compared with the uninoculated control plants. Inoculated TME 419 under well-watered conditions significantly increased the number of hyphae by 30.6% compared to cv. 60444 under the same well-watered condition.

**Table 1:** Frequency of AMF root colonization structures of micro-propagated cassava plantlets with and without AMF inoculation under well-watered and water stressed conditions at harvest (73 days).

Mean	Hyphae	Vesicles	Arbuscles	Root colonization frequency (%)
Inoculation (I)				
+AMF	59.7 ± 1.94a	14.1 ± 1.04a	6.91 ± 0.67a	83.8 ± 5.3a
-AMF	24.0 ± 1.35b	1.40 ± 0.43b	2.39 ± 0.64b	33.8 ± 5.7b
Water regime (W)				
Well watered (WW)	38.0 ± 1.83a	6.31 ± 1.18a	2.59 ± 0.70a	63.44 ± 5.3a
Water stressed (WS)	38.4 ± 1.64a	3.14 ± 0.43b	0.65 ± 0.65b	54.19 ± 5.7a
Variety (V)				
60444	39.4 ± 1.71a	8.41 ± 1.49a	2.54 ± 0.68b	61.66 ± 5.5a
TME-419	36.4 ± 1.76a	2.43 ± 0.62b	6.43 ± 0.71a	55.96 ± 5.5a
I*V				
+AMF*6044	56.3 ± 2.66a	19.41 ± 1.63a	5.59 ± 0.83	82.92 ± 3.22a
+AMF*419	63.3 ± 2.83a	3.41 ± 1.22c	0.59 ± 0.89	40.32 ± 2.45a
-AMF*60444	27.6 ± 2.01b	10.23 ± 1.19b	8.55 ± 1.05	78.40 ± 3.13a
-AMF*419	20.9 ± 1.79b	0.57 ± 0.28d	4.83 ± 0.89	26.60 ± 2.03
I*W				
+AMF*WW	62.4 ± 2.82a	13.46 ± 1.48a	7.50 ± 1.00	89.25 ± 7.5a
+AMF*WS	57.1 ± 2.68a	14.75 ± 1.36a	6.37 ± 0.89	78.37 ± 7.5a
-AMF*WW	22.8 ± 1.70b	2.95 ± 1.06b	1.05 ± 0.53	37.62 ± 7.5b
-AMF*WS	25.3 ± 2.13b	0.66 ± 0.33b	5.29 ± 0.82	30.22 ± 8.64b
I*W * V				
+AMF*WW*6044	52.0 ± 3.61bc	29.00 ± 2.69a	5.00 ± 1.88a	86.00 ± 4.64a
+AMF*WS*6044	61.0 ± 3.91ab	13.00 ± 1.80a	6.25 ± 1.25a	80.00 ± 4.47a
-AMF*WW*6044	20.2 ± 2.25e	17.50 ± 2.09a	4.00 ± 1.00a	42.0 ± 3.24a
-AMF*WS*6044	37.7 ± 3.54cd	0.60 ± 0.47a	0.33 ± 0.03a	38.7 ± 3.59a
+AMF*WW*419	75.0 ± 4.33a	6.25 ± 1.25a	11.25 ± 1.67a	92.5 ± 4.81a
+AMF*WS*419	53.5 ± 3.66bc	16.75 ± 2.04a	6.50 ± 1.27a	76.80 ± 4.38a
-AMF*WW*419	25.8 ± 2.54a	0.50 ± 0.35a	7.00 ± 1.32a	33.2 ± 2.88a
-AMF*WS*419	17.0 ± 2.38e	0.66 ± 0.47a	3.33 ± 1.05a	21.60 ± 2.67a

Mean values are shown in the table, means denoted with the same letters are not significantly different at p≤ 0.05, according to Tukey’s test.

**Table 2:** Biomass accumulation, total leaf chlorophyll, stable isotope C and N, total C and N, C:N and NUE of micro-propagated cassava plantlets with and without AMF inoculation under well-watered and water-stressed conditions at harvest (73days).

Mean	Belowground biomass (g)	Aboveground biomass (g)	d^13^C (°/_oo_)	d^15^N (°/_oo_)	Total C (%)	Total N (%)	C/N	NUE
Inoculation (I)								
+AMF	7.84 ± 0.98a	6.06 ± 0.59a	-28.76 ± 0.23a	10.23 ± 0.39b	47.23 ± 0.50a	2.69 ± 0.18b	18.64 ± 0.81a	9.36 ± 1.15a
-AMF	4.60 ± 1.01b	2.78 ± 0.61b	-29.00 ± 0.23a	11.75 ± 0.43a	47.89 ± 0.50a	3.68 ± 0.61a	14.62 ± 0.85b	5.26 ± 1.12b
Water regime (W)								
Well-watered (WW)	7.99 ± 0.99a	5.65 ± 0.59a	-28.91 ± 0.23a	10.67 ± 0.38a	47.80 ± 0.50a	3.24 ± 0.20a	15.76 ± 0.85a	9.24 ± 1.12a
Water stressed (WS)	4.44 ± 1.01a	3.20 ± 0.61b	-28.84 ± 0.23a	11.12 ± 0.43a	47.32 ± 0.50a	2.96 ± 0.18a	17.51 ± 0.81a	5.39 ± 1.15b
Variety (V)								
60444	4.31 ± 0.50a	4.31 ± 0.59a	-28.77 ± 0.23a	10.82 ± 0.41a	47.94 ± 0.50a	3.39 ± 0.19a	15.22 ± 0.85b	7.55 ± 1.14a
TME-419	4.53 ± 0.60a	4.53 ± 0.61a	-28.77 ± 0.23a	10.90 ± 0.41a	47.18 ± 0.50a	2.29 ± 0.19a	18.04 ± 0.83a	7.08 ± 1.12a
I*W								
+AMF*WW	10.92 ± 1.10a	7.90 ± 072a	-28.31 ± 0.30b	10.18 ± 0.56a	48.33 ± 0.66a	3.03 ± 0.26ab	20.56 ± 1.00a	12.82 ± 1.26a
+AMF*WS	4.75 ± 1.14b	4.23 ± 0.77b	-29.21 ± 0.30a	10.27 ± 0.56a	46.23 ± 0.66a	2.36 ± 0.26b	16.72 ± 1.00b	5.90 ± 1.26b
-AMF*WW	5.06 ± 1.148b	3.40 ± 0.77b	-28.51 ± 0.30ab	11.16 ± 0.56a	48.31 ± 0.76a	3.81 ± 0.30a	14.79 ± 1.11b	5.65 ± 1.26b
-AMF*WS	4.13 ± 1.14b	2.16 ± 0.77b	-29.37 ± 0.30a	11.98 ± 0.56a	47.47 ± 0.66a	3.55 ± 0.26ab	14.46 ± 1.00b	4.88 ± 1.38b

Mean values are shown in the table, means denoted with the same letters are not significantly different at p≤ 0.05, according to Tukey’s test.

For the number of vesicles, inoculation and water conditions interacted significantly (p<0.01) such that the inoculation of both varieties under water deficit conditions increased the number of vesicles by 78.9% over vesicle numbers in well-watered uninoculated plants. There was also a significant inoculation × variety (p<0.01) interaction. Inoculated cv.60444 had 82.4% more vesicles than the inoculated TME 419, this was also observed in the uninoculated varieties where cv. 60444 still had 94.4% more vesicles. For the arbuscules, there was a significant inoculation × water regime × variety (p ≤ 0.01) interaction. The number of arbuscules in the inoculated cv.60444 and TME 419 plants under water deficit were 94.7% and 48.7% higher than in the uninoculated plants of both varieties under the same water conditions.

### 3.2 Plant growth response during the acclimatization phase

Inoculation with *R. irregularis* at the beginning of acclimatization of cassava plantlets had a significant effect on the growth of micro-propagated cassava plantlets (**Supplementary Table S2**; **Table 3**). At the end of the 4 week acclimatization phase in the polypropylene trays (a week before the introduction of the water stress treatment), AMF inoculation led to a 60.7%, 50.2% and 57.0% increase in height (p<0.001) **Table 3**), stem diameter (p<0.001), and the number of green leaves (p<0.01), respectively. There was no effect of cassava variety (p>0.05) and no significant interaction between inoculation and variety (p>0.05).

**Table 3:** Plant height, stem diameter and number of fresh leaves of micro-propagated cassava plantlets with and without AMF inoculation during the acclimatization phase (before the introduction of water stress).

Mean	Plant height (cm)	stem diameter (mm)	No of leaves
Inoculation (I)			
+AMF	17.20 ± 1.65a	3.62 ± 0.28a	8.56 ± 0.72a
-AMF	10.70 ± 1.70b	2.41 ± 0.28b	5.64 ± 0.77b
Variety (V)			
60444	14.72 ± 1.67a	6.84 ± 0.74a	3.00 ± 0.27a
TME-419	13.14 ± 1.67a	7.42 ± 0.74a	3.00 ± 0.27b
I*V			
+AMF*60444	17.56 ± 1.90a	3.62 ± 0.34a	8.75 ± 1.02a
+AMF*419	16.76 ± 1.98a	3.63 ± 0.34a	8.38 ± 1.02a
-AMF*60444	11.84 ± 1.98a	2.48 ± 0.34a	4.86 ± 1.09a
-AMF*419	9.49 ± 1.98a	2.73 ± 0.34a	6.43 ± 1.09a

Mean values are shown in the table, means denoted with the same letters are not significantly different at p≤ 0.05, according to Tukey’s test.

### 3.3 Plant growth response at post-acclimatization after the introduction of water stress

**Figure 2:**
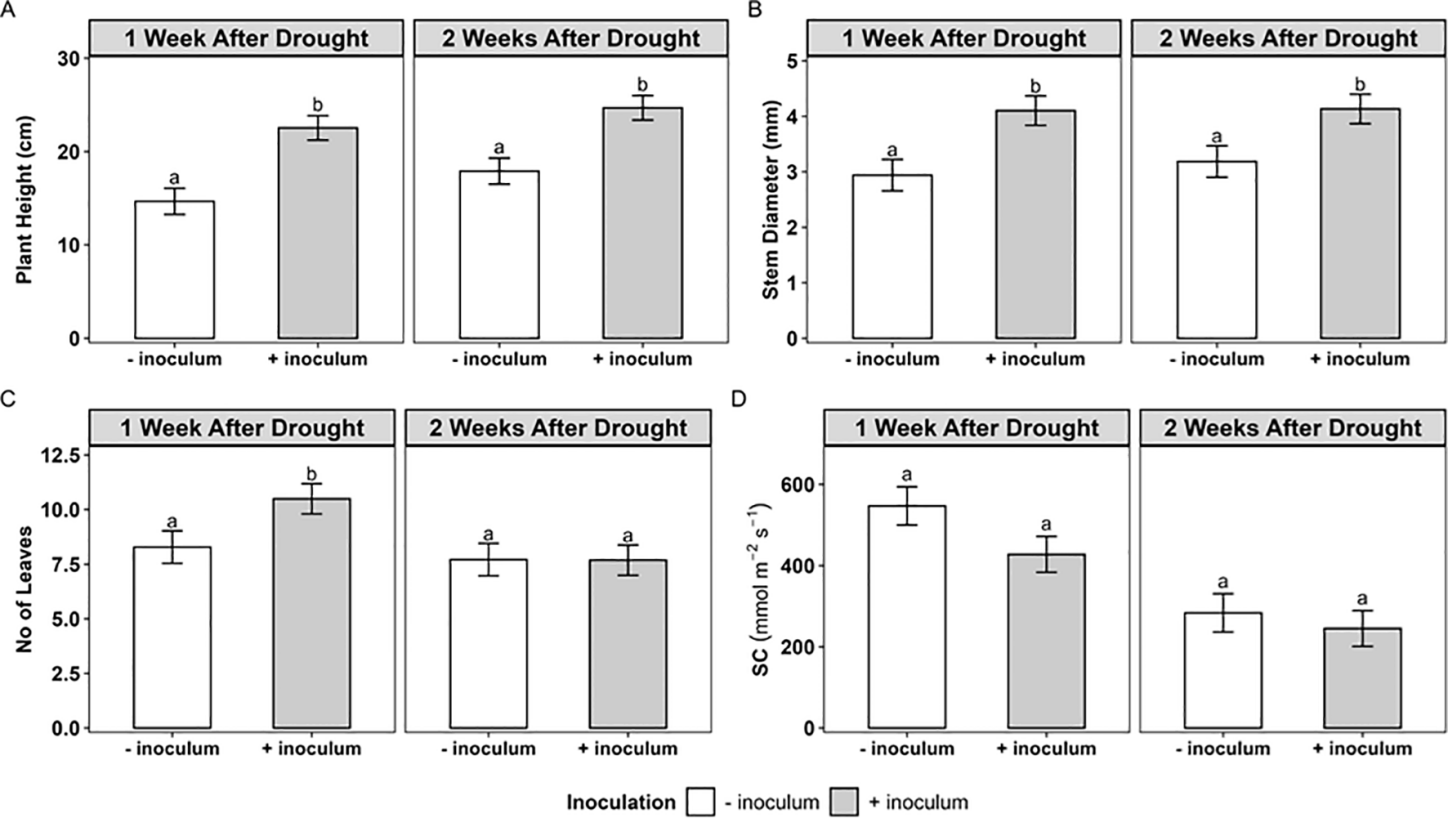
**(A)** Plant height, **(B)** stem weight **(C)** no of leaves and **(D)** stomatal conductance at 1 and 2 weeks after introduction of water stress (WAD) in micro-propagated cassava plantlets without AMF (- inoculum, white bars) or with AMF (+ inoculum grey bars) inoculation. The bars represent the mean ± SEM (*n* = 4). The bars represent the mean ± SE of n = 4 replicates. Bars sharing a common lowercase letter reveal no significant difference according to Tukey’s post hoc test at *p* < 0.05.

In the first week after the introduction of water deficit (Week 1), *R. irregularis* inoculation significantly improved plant height (+46.0%; p<0.001), stem mass (+ 29.4%; p<0.01) and the number of green leaves (+24.0%; p<0.01), compared with uninoculated plantlets (**Supplementary Table S3**; **Figure 2**). In the second week after the introduction of water deficit, inoculation significantly increased plant height (+25.5%; p<0.001) and stem diameter (+29.0%; p ≤ 0.05) (**Figure 2**), but the number of green leaves remained unaffected (**Supplementary Table S2**). There was no significant effect of the water regime or the variety,on plant growth parameters mentioned above, across these 2 weeks (**Supplementary Table S2**). Plant height had a significant inoculation × water regime interaction. Inoculated plants under water deficit were 14% taller than the uninoculated plantlets under water deficit, while under well-watered conditions inoculated plants were 48.6% taller than the uninoculated plants (**Figure 2**).

The stomatal conductance of inoculated plants was 20% lower than that of uninoculated plants at 1 WAD (p>0.05). The water regimes significantly affected the stomatal conductance in both weeks. In the first week after water deficit, the stomatal conductance declined from 557.3 mmol m^−2^ s^−1^ (well-watered) to 407.1 mmol m^−2^ s^−1^ (water deficit). This decline was aggravated in the second week of water deficit with 437.20 mmol m^−2^ s^−1^ (well-watered) and 56.1 mmol m^−2^ s^−1^ (water deficit) (p<0.001) being a difference of 87.1% (**Figure 2**.

### 3.4 Plant biomass accumulation and leaf chlorophyll content at harvest

**Figure 3:**
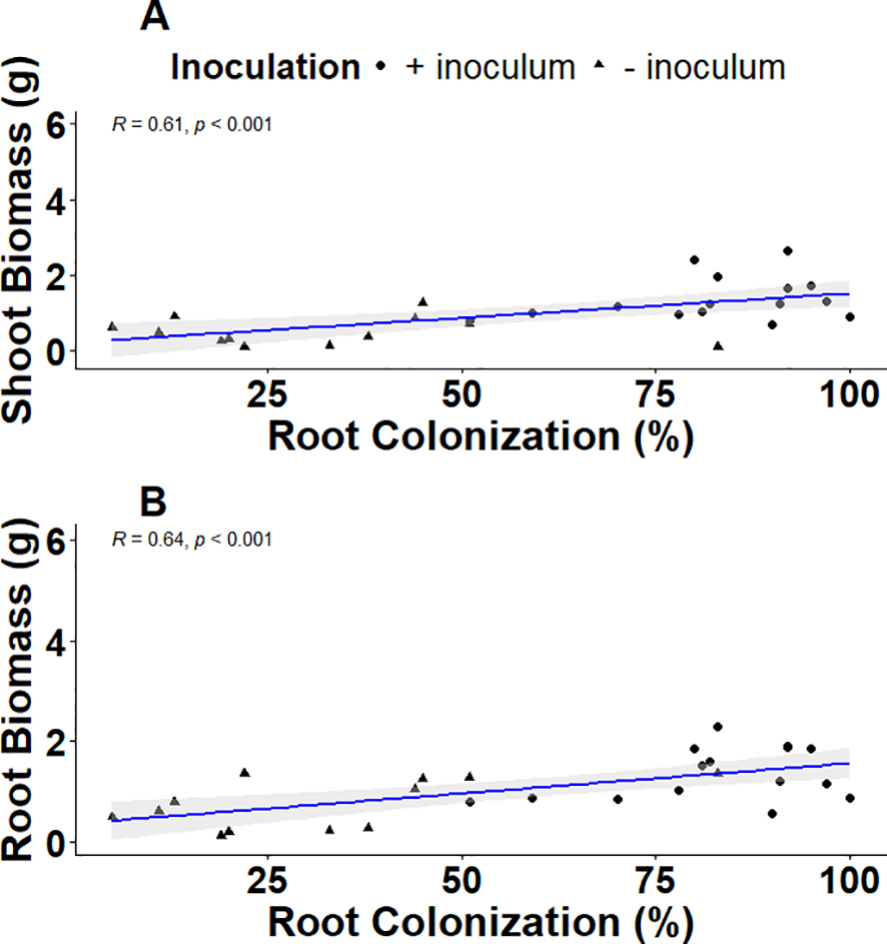
Linear relation between micro-propagated cassava **(a)** Shoot biomass), **(b)** Root biomass and mycorrhizal colonization levels (total mycorrhizal colonization). The shaded area shows 95% confidence intervals for the fitted line.

In both varieties, water deficit significantly reduced root biomass by up to 35.7% (p<0.001) and shoot biomass by 45.0% (p<0.01) compared to plants receiving sufficient water (**Supplementary Table S1**; **Table 2**). Inoculation with AMF had a significant impact on cassava biomass production leading to an increase of 63% in shoot and 68% in root biomass (both p<0.001). There was no significant inoculation x water regime interaction in shoot biomass (p>0.05), however root biomass had a significant interaction (p<0.001). The root biomass of well-watered cassava plants increased by 54.84% compared with uninoculated plants under the same water condition. The inoculated and uninoculated water-deficit plants had a 56.0% and 58.0% lower root biomass than the inoculated well-watered plants, respectively. The shoot biomass (R = 0.57, p=0.002) and the root biomass (R = 0.59, p=0.001) were significantly positively correlated with total root colonization (**Figure 3**).

**Figure 4:**
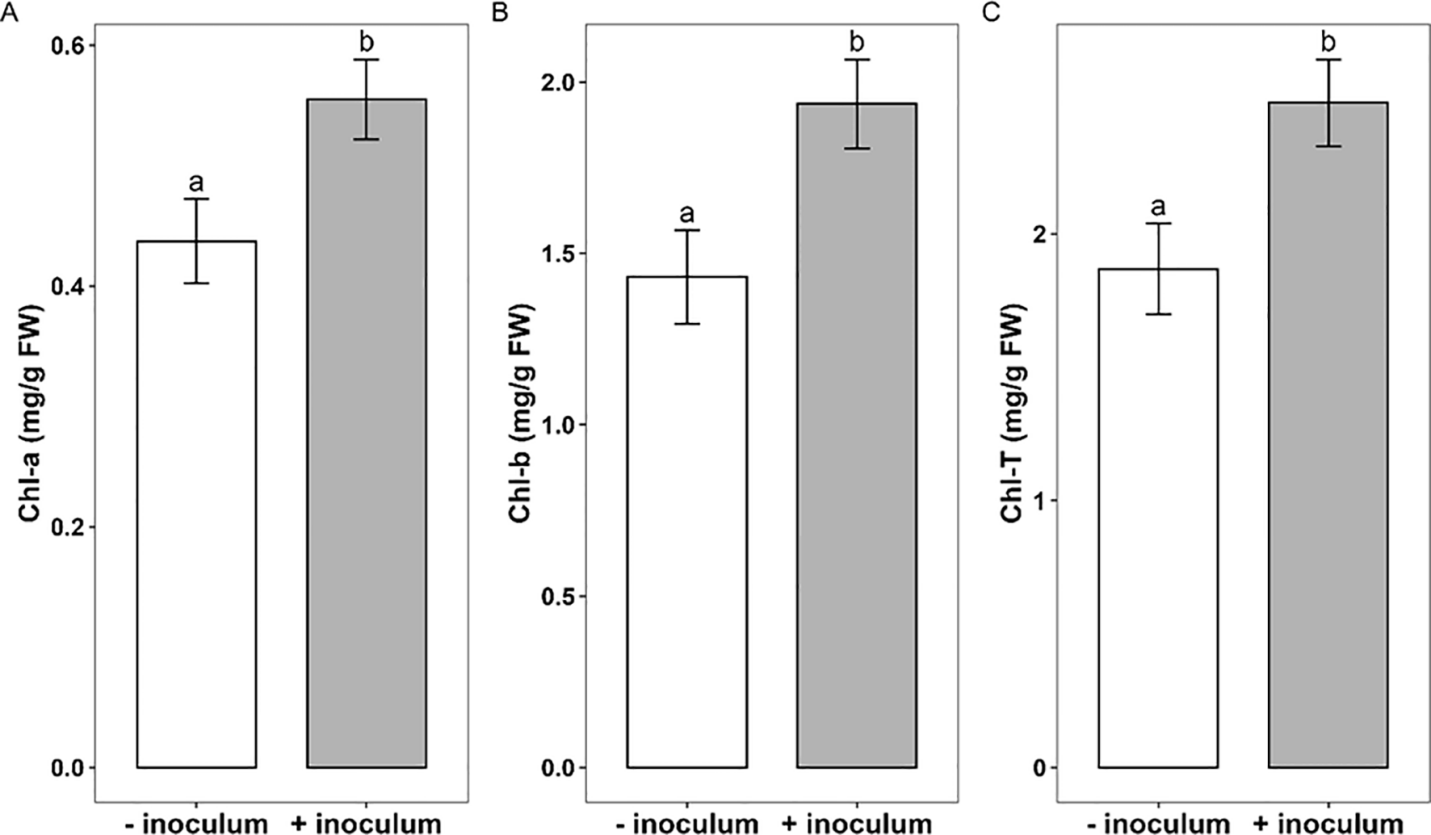
**(A)** Chlorophyll a (Chl *a*), **(B)** Chlorophyll b (Chl *b*) and **(C)** Total chlorophyll (Chl *a*+*b*), of cassava plantlets fresh leaves without AMF (- inoculum, white bars) or with (+ inoculum, grey bars). The bars represent the mean ± SE of n = 4 replicates. Bars sharing a common lowercase letter reveal no significant difference according to Tukey’s post hoc test at *p* < 0.05.

Chlorophyll a (Chl *a*) increased by 21.6%, chlorophyll b (Chl *b*) by 25.5% and the total chlorophyll (Chl *a*+*b*) increased by 24.9% in plants inoculated with AMF (**Figure 4**). The different water regimes and cassava varieties had no effect, and there were no significant interactions of all tested factors (p>0.05).

### 3.5 Nitrogen use efficiency

The nitrogen use efficiency of micro-propagated cassava was significantly increased by 44.2% when inoculated (p<0.001) (**Supplementary Table 1**; **Table 2**). Well-watered plantlets had higher NUE compared with plants subjected to water deficit (p>0.05). There was no significant difference in the NUE between the varieties. However, there was a significant inoculation × water regime interaction (p<0.01): the NUE of well-watered inoculated plantlets was 54.4% higher than that of well-watered plants without inoculum, while plantlets under water deficit conditions had no significant difference in NUE whether inoculated or not (**Table 2**).

### 3.6 Stable isotopes of C and N, tissue percent C and N, C:N

**Figure 5:**
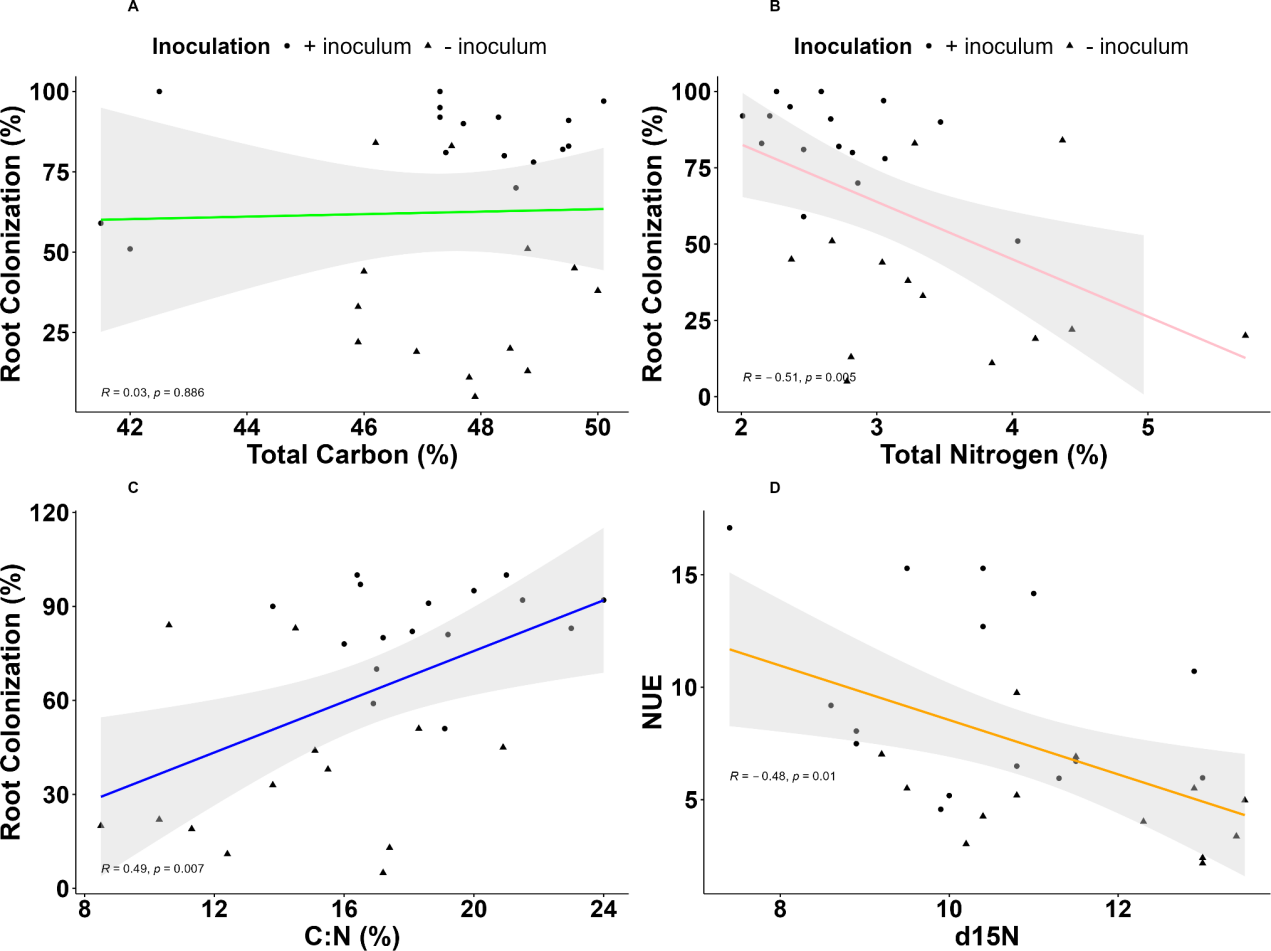
Linear relations between **(a)** Total carbon, **(b)** Total nitrogen, **(c)** C:N ratio **(d)** Leaf δ^15^N (‰) and total mycorrhizal colonization of micro-propagated cassava (total mycorrhizal colonization, arbuscule colonization) of AMF inoculated micro-propagated cassava. The shaded area shows 95% confidence intervals for the fitted line.

*R. irregularis* inoculation had no significant effect on the δ ^13^C in the YFEL of cassava plantlets. However, there was a significant inoculation × water regime interaction (p<0.01). Either inoculated or not, the plants under water deficit had less negative δ^13^C (-3.40%_)_ when compared to the inoculated and uninoculated well-watered plants. The C concentration in the plant tissue did not respond to any treatment and there were no significant interactions (**Table 2**). Tissue C content was not significantly correlated with total root colonization (**Figure 5A**). Inoculation significantly decreased the N concentration in the plant tissue (-26.9%; p<0.05) compared with uninoculated plantlets. The water regimes and varieties had no significant effect on N concentration (**Table 2**) and there was no significant interaction (p>0.05). There was a negative correlation (R=-0.51, p= 0.005) (**Figure 5B**) between N concentration with total root colonization. The δ^15^N in the YFEL of the micro-propagated cassava plants was 12.97% lower compared with the uninoculated plants (**Supplementary Table 1**; **Table 2**). All other treatments and interactions were not significant. There was a negative correlation (R=-0.48, p= 0.01) (**Figure 5D**) between δ ^15^N and NUE.

The C:N increased by 21.5% in inoculated compared with uninoculated plantlets (**Table 2**). The C:N of TME 419 was higher than that of cv.60444 (+15.6%; p<0.01). There was a significant inoculation × water regime interaction. The C:N of the inoculated well-watered plants was the highest compared with other treatments (p<0.05). The C:N of the inoculated well-watered plants was increased by 28.0% compared with the uninoculated well-watered plants, and by 18.8% and 29.6% compared with the inoculated and uninoculated water deficit plants. We observed a positive correlation (R = 0.49, p= 0.007) (**Figure 5C**) between C:N and colonization intensity.

### 3.7 FAMD of cassava morphological and physiological responses to inoculation and water stress

**Figure 6:**
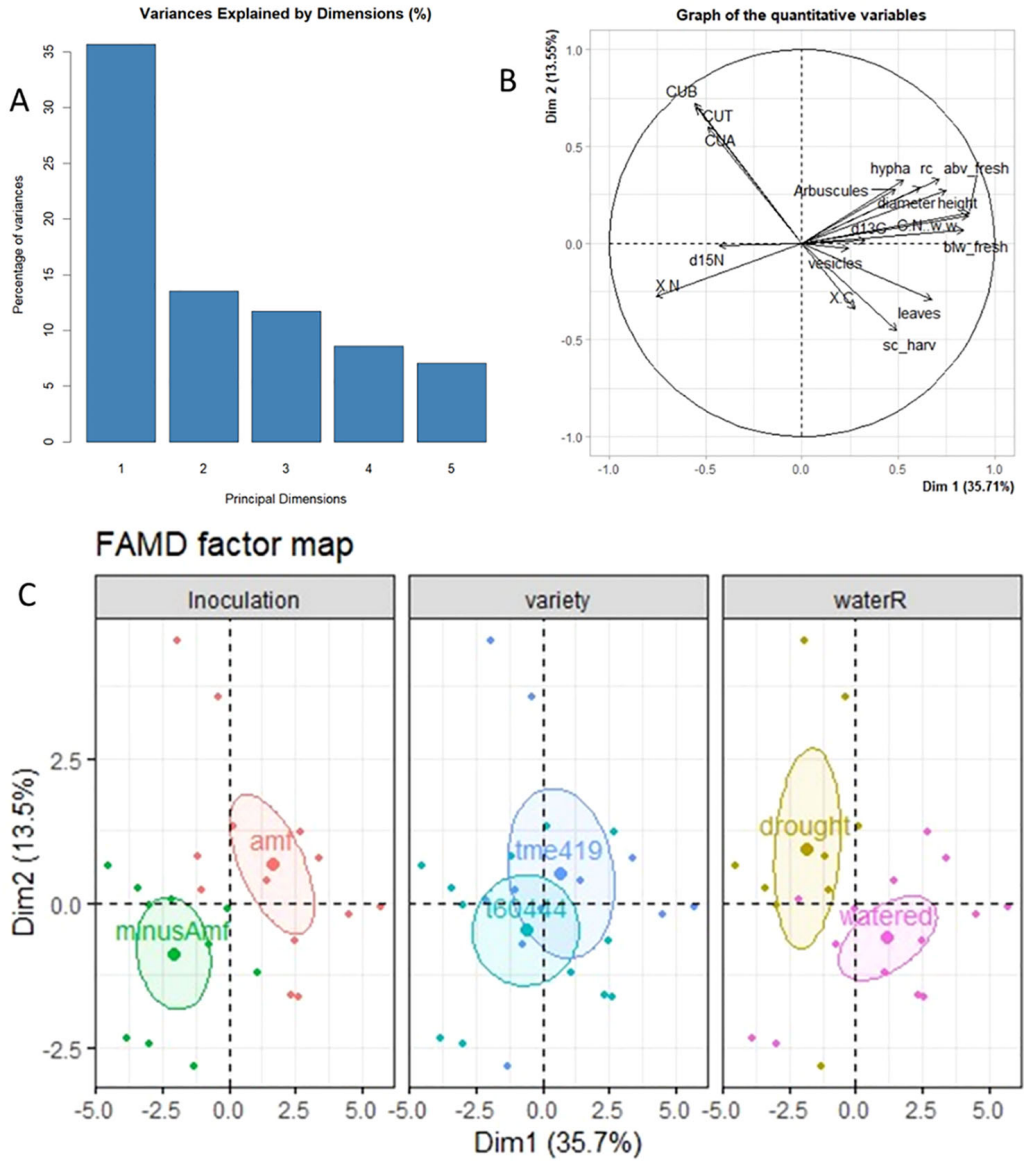
Factorial Analysis of Mixed Data (FAMD) plots of growth and physiological characteristics of two varieties of micropropagated cassava with or without commercial *R. intraradices* under two water regimes; **(A)** variance explained by principal dimensions **(B)** biplot of quantitative variables **(C)** factor map of individual samples with indication of the treatments inoculation, variety and water regime. Ellipses denote 95% confidence interval. rc = root colonization %; abv_fresh, above ground biomass (fresh); blw-ground, below-ground biomass (fresh); sc_harv, stomatal conductance; CUA, chlorophyll a; CUB, chlorophyll b; CUT, total chlorophyll; X.C, total carbon (leaf); X.N, total nitrogen (leaf); C:N.w.w, C:N ratio.

The cassava plantlets’ NUE, above-ground biomass, C:N, below-ground biomass, total nitrogen (TN) concentration, root colonization %, no of leaves and stem diameter were the key contributors to the first principal axis of the FAMD, while total chlorophyll, stomatal conductance, δ ^13^C, total chlorophyll, and δ ^15^N were the main correlates with the second principal axis (**Figure 6a**; **Supplementary Figure S1**). The analysis further indicates that the number of leaves, stomatal conductance, total carbon % and vesicles were all positively correlated but had an inverse relationship with the leaf chlorophyll content. Plant growth, shoot and root biomass, hyphae, arbuscules, total root colonization %, δ ^13^C, and C:N were all positively correlated. However, all these parameters had a negative correlation with δ ^15^N and total nitrogen% (**Figure 6b**). **Figure 6c** shows that the groups with or without AMF inoculation were different, the varieties responded similarly, while the water regime also led to distinctions between the groups.

## 4 Discussion

This work provides new information on morphological and physiological adjustments to water stress during acclimatization and post-acclimatization of micropropagated cassava colonized by AMF under realistic unsterilized soil conditions. Our experiment demonstrated clearly that the inoculation with commercial *R. irregularis* enhanced the growth of micropropagated cassava. AMF support plant growth, however the benefits were substantially greater under well-watered than under water deficit. Additionally, it revealed that the micropropagated cassava receiving the given inoculum had lower nitrogen concentration due to the improved NUE stimulated by enhanced biomass production.

### 4.1 Mycorrhizal colonization and plantlet growth

The rate at which AMF colonize plant roots is an indicator of successful symbiotic engagement between host plants and the fungi (Wang et al., 2021). In the plantlets that were subjected to inoculation, it was observed that the AMF not only colonized the roots through hyphae but also formed vesicles and arbuscules. The total root colonization estimated 5 weeks after the ex vivo transfer was 81%, of which 60% had hyphae, 14% vesicles and 7% arbuscules. The presence of the three structures indicates the successful formation of a functioning symbiotic association because arbuscules are the principal structures that allow nutrient exchange whereas vesicles function as reproductive organs and store carbohydrates (Harrison, 2005; Parniske, 2008; Harrison, 2005). Because previous studies have shown that commercial AMF inocula may not always be compatible with host plants or may not be able to compete with native soil AMF communities, the establishment we discovered is a crucial result (Berruti et al., 2016; Vosátka et al., 2012). We also observed that the native AMF species in the unsterilized natural soil used in this experiment colonized 33% of the cassava roots. This indicates that the used soil had compatible AMF, yet they were less effective in colonization than the commercial product due to differences in spore density and biogeographic factor (references).

At the end of the acclimatization phase (8 WAP), we found that inoculated plantlets were taller, had larger stem diameters and had more green leaves. The enhanced growth observed in the acclimatization phase was sustained through the post acclimatization period. The growth of the micro-propagated cassava plantlets was positively influenced by a higher root colonization rate, comparable to the reported effects in micro-propagated strawberry plants (El Meskaoui et al., 2008). The similar response to AMF colonization found in both varieties suggests that both cv. 60444 and TME 419 have a similar compatibility with R. irregularis. In a field trial in Colombia, the cassava cultivars MCOL2737 and COL2215 showed a colonization rate of more than 50% (Ceballos et al., 2013). Thanni et al. (2023) showed that an AMF inoculum consisting of multiple AMF taxa increased the rate of root colonization by 50%, compared with inoculation with Rhizophagus irregularis only in TME 419 in a screen house. More research is necessary to ascertain if the beneficial effects of inoculating with *R. irregularies* on cassava growth during and post acclimatization can also be observed in other cassava varieties, and if other AMF species can cause similar positive effects since the responsiveness of crops to inoculation is dependent on both AMF species and crop species (Aliyu, 2019; Van Geel et al., 2016).

### 4.2 Influence of AMF on cassava biomass accumulation under varying water conditions

Under well-watered conditions, AMF inoculation doubled the plant biomass, suggesting AMF enhanced nutrient uptake when water is not limiting. Usually, the effect size of AMF inoculation on plant biomass increases with AMF colonization rate (Oyetunji et al., 2023; Peña et al., 2020), as even in unfavorable conditions the AMF hyphae assist the host plants by increasing the root surface thereby enabling it to gain access to water, micro and macronutrients (Ndeko et al., 2019; Otun and Ikechukwu, 2022). While AMF have been reported to enhance biomass production even under water deficit in some micropropagated systems; for example *Glomus* sp. increased shoot and biomass of micropropagated strawberry following short term water withdrawal (Borkowska, 2002). However in our study under the water deficit condition, AMF inoculation did not translate into increased biomass, suggesting that drought constrained the functional benefits of the symbiosis and that AMF-mediated growth enhancement in cassava was strongly dependent on adequate water availability.

### 4.3 Influence of AMF inoculation on leaf stomatal conductance and chlorophyll content at varying water conditions

Water deficit reduced the stomatal conductance of the plantlets consistently during both weeks of water deficit. In fact, by the 2^nd^ week, there was a drastic decline in stomatal conductance. Contrary to our expectation, an increased colonization rate did not help alleviating the stress imposed by reduced stomatal conductance in inoculated plantlets. This finding contradicts some reports indicating that AMF can increase stomatal conductance in various plants under water deficit conditions (Augé et al., 2015; Gong et al., 2013; Li et al., 2019). However, our result corroborates those of Peña Venegas et al. (2021) who showed that stomatal conductance of cassava inoculated with different *R. irregularis* isolates varies, and even can be lower in inoculated plants under drought conditions.

Differences in the quantities of chlorophyll may indicate underlying functional changes to plants’ photosynthetic capacity (Díaz et al., 2016; Esteban et al., 2015) since the synthesis of chlorophyll depends on the mineral nutrition of the plant (Bojović and Stojanović, 2005). We found that the inoculation with AMF improved the total leaf chlorophyll content of micropropagated cassava by 25% compared to uninoculated plants. However, the chlorophyll content response to drought was the same with that under well-watered conditions. This suggests that the increment observed in chlorophyll content after inoculation in this study is not a drought tolerance mechanism as suggested by Bouskout et al. (2022) but rather the result of the increased growth as observed in the inoculated treatment. This corroborates the finding of Estrada-Luna and Davies (2003) who recorded a substantial increase in chlorophyll during post-acclimatization of *Capsicum annuum* inoculated with AMF. Furthermore, Liu et al. (2015), and Balestrini et al. (2020) demonstrated that mycorrhizal plants typically have a greater photosynthetic capability than non-mycorrhizal plants. The AMF-colonized plant can experience these benefits through increased transpiration rate, photosynthetic rate, and chlorophyll content (Chandrasekaran et al., 2019).

### 4.4 Influence of AMF inoculation on stable isotopes of C and N in micropropagated cassava during varying water conditions

We initially expected that water withdrawal would reduce stomatal conductance and consequently result in higher (less negative) δ^13^C due to reduced discrimination against 13CO2, as predicted by classical isotope theory (Farquhar et al., 1989; Santesteban et al., 2012). Contrary to this expectation, the plants exposed to water deficit exhibited more negative δ^13^C values, irrespective of the AMF inoculation. Importantly, this pattern occurred despite the absence of a consistent reduction in stomata conductance under drought conditions, indicating that variations in δ^13^C could not be directly attributed to stomatal regulation in this study. This lack of correspondence between δ^13^C and stomatal conductance suggest a decoupling driven by post-stomatal controls on photosynthesis, such as changes in mesophyll conductance and biochemical limitation under drought (Seibt et al., 2008; Cernusak et al., 2013). The δ ^15^N in inoculated plants was lower than that of the uninoculated plants even though inoculation increased plant biomass. The enhanced NUE of the inoculated plants could have led to a reduced isotopic fractionation during the process of nitrogen absorption, leading to a lower δ ^15^N because changes in δ^15^N may be indicative of high N utilization in plants (Vallano and Sparks, 2013).

### 4.5 Mycorrhizal colonization on plants percent C, N and NUE in micro-propagated cassava

AMF inoculation increased both root and shoot biomass, particularly under well-watered conditions, indicating enhanced carbon assimilation and growth. Despite this, AMF-inoculated plants exhibited lower tissue nitrogen concentrations and higher C:N ratios than uninoculated plants. This pattern reflects a classic nitrogen dilution effect, whereby nitrogen acquired by the plant is distributed across a larger biomass pool as growth accelerates (Jarrell and Beverly, 1981; Marschner, 2012). Importantly, this reduction in tissue nitrogen concentration occurred alongside a marked increase in nitrogen use efficiency (NUE), demonstrating that AMF-inoculated plants produced substantially more biomass per unit nitrogen absorbed.

Beyond biomass dilution, several mechanistic pathways likely explain the observed improvement in NUE. Arbuscular mycorrhizal fungi enhance nitrogen acquisition by extending the effective absorptive surface area of the root system through extraradical hyphae, thereby accessing nitrogen pools beyond the root depletion zone (Hodge et al., 2001; Smith and Read, 2008). AMF colonization can also modify root architecture, increasing root branching and functional root length, which further improves nitrogen uptake efficiency (Berta et al., 2005; Gutjahr & Parniske, 2013). In addition, AMF may indirectly influence nitrogen availability by altering rhizosphere microbial communities and stimulating nitrogen mineralization processes (Hodge and Fitter, 2010; Veresoglou et al., 2012). Together, these mechanisms enhance nitrogen acquisition and utilization efficiency, supporting rapid biomass accumulation. However, because biomass production outpaces nitrogen concentration, nitrogen becomes diluted within plant tissues, resulting in lower %N but higher NUE.

Considering the possibility that nitrogen might be a limiting factor since AMF normally supply a substantial amount of phosphorus to plants, there would be need to provide adequate N to plant during the pre and post acclimatization phase that extends beyond that of our study (41 days), so as not to lose out on the initial benefit provided by the fungi.

## 5 Conclusion

Our study shows that including *R. irregularis* inoculum during the first phase of cassava acclimatization yields remarkable positive results in terms of root colonization, growth, physiological response, and nitrogen use efficiency (NUE) under well-watered conditions. Therefore, we suggest to employ AMF inoculation during the acclimatization phase to aid in the pre-adaptation of micropropagated cassava before being transferred to the field. Nevertheless, it is important to acknowledge that the fast increase in biomass resulted in a decrease in nitrogen concentration in plants, and if left unattended, the dilution effect might have the potential to hinder plant growth by causing nutritional deficit of N. Hence, doing more research on varying sources and levels of N fertilization relating to AMF would enable the development of more effective management techniques for nitrogen (N) delivery that align with the substantial N requirements of inoculated plants, thereby maximizing the advantages of The important results NUE in this study provide crucial information for future AMF investigation, as most of the previous research on AMF has emphasized its effects on phosphorus.

## Data Availability

The original contributions presented in the study are included in the article/**Supplementary Material**. Further inquiries can be directed to the corresponding author.
