# Supplementary material for: Enhanced morphological and physiological responses of micro-propagated cassava through arbuscular mycorrhizal fungus inoculation
Source: Front Plant Sci. 2026 Mar 9;17:1692288. doi: 10.3389/fpls.2026.1692288 (PMC13006646; doi:10.3389/fpls.2026.1692288)
Supplement: Supplementary file 1 [file DataSheet1.docx]

**SUPLEMENTARY INFORMATION**

**TABLES**

**S Table 1:** ANOVA F-values and significances (Linear Mixed Effect Model, LMEM) for biomass accumulation and total leaf chlorophyll, stable isotopes of C and N, and NUE of cassava plantlets and ANOVA chisq-values and significances (Generalized Linear Model, GLM) for the response of root colonisation structures, with and without inoculum subjected to two levels of water stress conditions in the greenhouse.

|  | **Shoot biomass**  **(g)** | **Root biomass**  **(g)** | **Total leaf chlorophy content (%)** | **Hyphae (%)** | **Vesicles (%)** | **Arbuscule (%)** | **AMF Total Colonization (%)** | **d^13^C**  (^o/oo^) | **d^15^N**  (^o/oo^) | **% C** | **%N** | **C/N** | **Nitrogen use efficiency** |
| --- | --- | --- | --- | --- | --- | --- | --- | --- | --- | --- | --- | --- | --- |
| **Inoculation (I)** | 25.08*** | 18.79*** | 5.21^*^ | 225.86*** | 88.56*** | 16.45*** | 322.4*** | 0.60 | 13.29* | 1.03 | 13.29* | 21.63*** | 24.92*** |
| **Water Regime (W)** | 13.98** | 22.67*** | 0.15 | 0.32 | 19.00*** | 9.16** | 10.74** | 0.08 | 1.52 | 0.53 | 1.52 | 4.11 | 24.22** |
| **Variety (V)** | 0.11 | 0.64 | 0.24 | 0.36 | 63.75*** | 13.08*** | 3.08 | 0.43 | 0.16 | 1.37 | 0.16 | 10.63** | 0.37 |
| **I*W** | 3.38 | 12.23** | 1.97 | 2.92 | 20.77*** | 5.07* | 1.09 | 7.71^**^ | 0.99 | 4.05 | 0.99 | 5.84* | 13.50** |
| **I*V** | 0.01 | 0.39 | 0.44 | 8.51** | 9.99** | 0.57 | 13.98*** | 0.02 | 2.13 | 5.77* | 2.13 | 1.04 | 0.06 |
| **W*V** | 0.53 | 2.42 | 0.004 | 32.44*** | 48.59*** | 0.90 | 3.35 | 1.95 | 0.07 | 0.40 | 0.07 | 0.08 | 0.27 |
| **I*W*V** | 1.23 | 0.04 | 0.08 | 4.35* | 1.97 | 6.73** | 1.23 | 0.002 | 1.75 | 8.19** | 1.75 | 0.62 | 0.31 |
|  |  |  |  |  |  |  |  |  |  |  |  |  |  |

F Values are shown in the table, significance in this table was based on a p level of *p ≤ 0.05; **p< 0.01; ***p<0.001.

**S Table 2:** ANOVA F-values and significances (Linear Mixed Effect Model, LMEM) for the responses of plant height, stem diameter and no of fresh leaves of micro-propagated cassava plantlets with and without inoculum during the acclimatisation phase (before the introduction of water stress).

| **Treatments** | **Plant height (cm)** | **Stem diameter (mm)** | **No of green leaves** |
| --- | --- | --- | --- |
| **Inoculation (I)** | 21.71*** | 14.47*** | 7.66** |
| **Variety (V)** | 1.22 (0.28) | 0.01(0.91) | 0.28 (0.62) |
| **I*V** | 0.31(0.57) | 0.02 (0.86) | 0.85 (0.36) |

F values are shown in the table, significance in this table was based on a p level of *p ≤ 0.05; **p< 0.01; ***p< 0.001.

**S Table 3:**ANOVA F-values and significances (Linear Mixed Effect Model, LMEM) for the responses of plant height, stem diameter, no of fresh leaves and stomatal conductance of micro-propagated cassava plantlets with and without *R. irregularis inoculum* after 1 and 2 weeks after the introduction of water stress in a greenhouse condition.

|  | **Height** | | **Stem diameter** | | **No of green leaves** | | **Stomatal conductance**  **(**mmol m^−2^ s^−1^**)** | |
| --- | --- | --- | --- | --- | --- | --- | --- | --- |
|  | 1WAD | 2WAD | 1WAD | 2WAD | 1WAD | 2WAD | 1WAD | 2WAD |
| **Inoculation(I)** | 21.40 *** | 16.04*** | 1.22** | 4.59* | 10.73** | 0.00 | 9.75*** | 1.32 |
| **Water regime (W)** | 0.001 | 2.22 | 0.01 | 1.31 | 0.62 | 2.90 | 1.58*** | 15.09*** |
| **Variety (V)** | 0.001 | 0.51 | 0.73 | 0.12 | 2.08 | 0.48 | 0.43 | 0.95 |
| **I*W** | 6.84** | 5.44* | 2.02 | 0.78 | 1.58 | 0.75 | 0.45 | 0.00 |
| **I*V** | 0.001 | 0.31 | 0.85 | 1.79 | 0.01 | 0.00 | 0.40 | 0.15 |
| **W*V** | 1.37 | 1.36 | 13.40 | 0.16 | 2.46 | 1.65 | 1.72 | 0.00 |
| **I*W*V** | 0.15 | 0.06 | 0.26 | 0.82 | 1.48 | 0.44 | 3.24 | 0.01 |

F values are shown in the table, significance in this table was based on a p level of *p ≤ 0.05; **p< 0.01; ***p< 0.001.

**FIGURES**


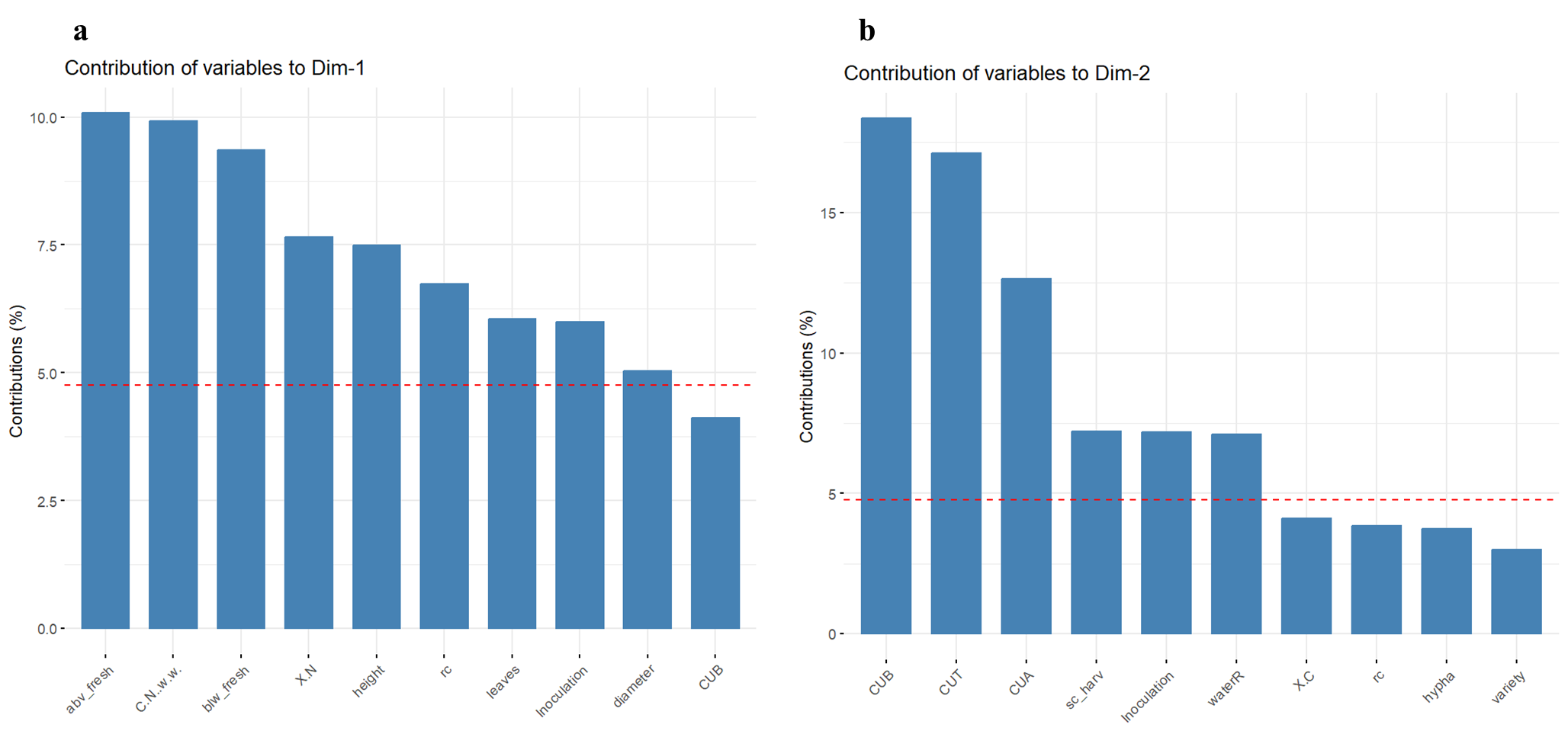


**S Figure 1**: The contributions of variables for dimensions 1 and 2 of the Factorial Analysis of Mixed Data (FAMD) plots on growth and physiological characteristics of two varieties of micro-propagated cassava with or without commercial *R. irregularis* under two water regimes for; (A) variance explained by principal dimensions (rc = root colonization %; abv_fresh = above ground biomass (fresh), blw-ground = below-ground biomass (fresh); sc_harv = stomatal conductance; CUA = chlorophyll a; CUB = chlorophyll b; CUT= total chlorophyll; X.C = total carbon (leaf); X.N = total nitrogen (leaf); C:N.w.w = C:N.
